# Association between gross motor function and postural control in sitting in children with Cerebral Palsy: a correlational study in Spain

**DOI:** 10.1186/s12887-015-0442-4

**Published:** 2015-09-16

**Authors:** Sergio Montero Mendoza, Antonia Gómez-Conesa, María Dolores Hidalgo Montesinos

**Affiliations:** Research Group in Physiotherapy and Health Promotion, Regional Campus of International Excellence “Campus Mare Nostrum”, Murcia University, Murcia, Spain; Department of Physiotherapy, Faculty of Medicine, University of Murcia, 30100 Espinardo-Murcia, Spain

**Keywords:** Cerebral palsy, Seating, Movement disorders, Postural balance, Measurement

## Abstract

**Background:**

Cerebral palsy (CP) is one of the causes of physical disability in children. Sitting abilities can be described using the Level of Sitting Scale (LSS) and the Gross Motor Function Classification System (GMFCS). There is growing interest in the sitting posture of children with CP owing to a stable sitting position allows for the development of eye-hand coordination, functions of the upper extremities and functional skills. Besides, in recent years researchers have tried to develop a new terminology to classify the CP as performed by the Surveillance of Cerebral Palsy in Europe (SCPE), in order to improve the monitoring of the frequency of the PC, providing a framework for research and service planning. The aim of this study was to analyse the relationship between GMFCS and LSS. The second purpose was to describe how the SCPE relates to sitting abilities with the GMFCS and LSS.

**Methods:**

The study involved 139 children with CP (range 3–18 years) from 24 educational centres. Age, gender, CP classification according to SCPE, GMFCS and LSS levels were recorded by an experienced physiotherapist.

**Results:**

A significant inverse relationship between GMFCS and LSS score levels was found (r_s_ = −0.86, *p* = 0.00). 45.3 % of the children capable of leaning in any direction and of re-erecting the trunk (level VIII on the LSS) could walk without limitation (level I on the GMFCS). There were differences in the distribution of the GMFCS (*χ*^2^(4):50.78) and LSS (*χ*^2^(7): 37.15) levels and CP according to the distribution of the spasticity (*p* <0.01).

**Conclusions:**

There was a negative correlation between both scales and a relation between sitting ability and the capacity to walk with or without technical devices. GMFCS and the LSS are useful tools for describing the functional abilities and limitations of children with CP, specially sitting and mobility. Classification based on the distribution of spasticity and the gross motor function provides clinical information on the prognosis and development of children with CP.

## Background

Cerebral palsy (CP) is one of the causes of physical disability in infants. It is described as a group of permanent disorders that affect the development of movement and posture and attributed to non-progressive disorders in the fetal development or infant brain [1]. The problems associated with movement and posture include abnormal muscle tone, activity limitation, lack of equilibrium and alterations in the alignment that affect sitting position favouring the appearance of compensatory postures in the three cardinal planes [1, 2]. Because of the motor impairments of the trunk and limbs, there is an inability to generate force to maintain antigravity postural control, thus leading to abnormal posture. Postural control affects not only sitting and standing but also the ability to sequence the movements appropriately [3].

There is growing interest in the sitting posture of children with CP owing to a stable sitting position allows for the development of eye-hand coordination, functions of the upper extremities, functional skills and self-care, cognitive development and social interaction [4, 5]. Sitting ability is analysed to detect whether the child is adopting asymmetric postures that favour shortening of the soft tissues and the appearance of deformities. For these reasons, the physiotherapists need reliable and assessment measures for sitting with high levels of responsiveness and validity that would permit effective treatment strategies [6].

One method to classify sitting abilities in children with neuromotor disorders is the Level of Sitting Scale (LSS). The LSS was designed by a team of clinicians and researchers at Sunny Hill Health Centre for Children [7]. The LSS consists of eight levels based in the amount of support required to maintain the sitting position and, in the case of children who can sit independently without support, the stability of the child while sitting. The levels range from level I (unable to sit for 30 s with one person assisting) to level VIII (able to sit independently for 30 s and move in and out of base of support in four directions). Fife et al. [8] documented the LSS interrater and test- retest reliability. The LSS reliability estimates were fair to good. Roxborough et al. [7] suggest LSS may be useful for evaluative purposes, in addition to its role as a classification index.

Sitting posture control and the severity of the disability in the daily lives of children with CP can also be described using the Gross Motor Function Classification System (GMFCS) [8]. The GMFCS includes five levels and five age bands. Level I represents children with the most independent motor function and level V represents children with the least. The GMFCS was developed to provide a standardized classification of the patterns of motor disability in children with CP aged 1 to 18 years [9, 10]. The GMFCS is based on self-initiated movement, with emphasis on sitting, transfers, and mobility. The focus is to determine the level that best reflects the present abilities and limitations of the child and youth in relation to gross motor functions. The reliability of GMFCS has been documented (interrater reliability of 0.75 and reliability of 0.93). A good predictive validity has been reported for children over the age of 2 years [11]. The authors of the scale conclude it is a useful tool for communication between professionals, for making clinical decisions and for research [8].

The ability to acquire the postural control in sitting will influence in the development of other gross motor functions such as standing and walking. In clinical practice, both scales (LSS and GMFCS) are used to evaluate the sitting abilities of children with CP, including their sitting posture control, and also to evaluate the effectiveness of certain treatments such as the use of adapted seating [3].

These two classification systems were based on the International Classification of Functioning, Disability and Health (ICF). Nevertheless, whereas the authors of the GMFCS were interested in the distinction between capability, performance and the perspective that environmental and personal factors influence in the performance of gross motor function, the LSS was associated with the component of activity of the ICF and the relationship between sitting ability and the amount of postural support adaptations needed for children with neuromotor disorders. The LSS has potential to assist therapist in determining what level of external postural support is required to maintain a sitting position [6]. Chung et al. [3] support the use of the LSS and GMFCS in clinical research to enable comparisons across the studies in terms of motor severity.

Besides, in recent years researchers have tried to develop a new terminology to classify the CP due to the clinical complexity that results from the topographic classification or motor impairment, as performed by the Surveillance of Cerebral Palsy in Europe (SCPE), in order to improve the monitoring of the frequency of the CP, providing a framework for research and service planning [12, 13]. The classification of CP should be based on CP type and motor function. The sitting ability is a strong predictor for ambulation in children with CP at 2 years of age. Therefore, the knowledge of sitting ability is relevant to predict future ability in these children [14, 15].

To attempt to understand the clinical relationship between the gross motor function and sitting abilities, the aim of this study was to analyse the relationship between the GMFCS and LSS in children with CP. The second purpose was to describe how the classification of CP according to the Surveillance of Cerebral Palsy in Europe (SCPE) relates to sitting abilities with the GMFCS and LSS.

## Methods

The study was carried out in educational centres in Murcia (Spain) from January to June in 2013.

### Inclusion criteria

The inclusion criteria included children aged 3–18 diagnosed with CP in educational centres, regardless of educational level.

**Fig. 1:**
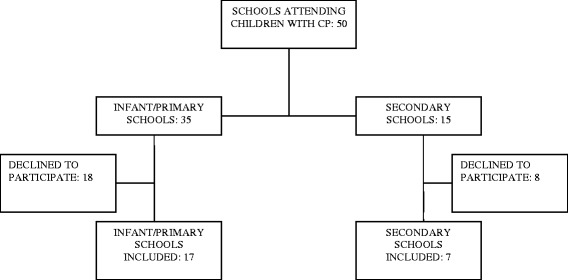
Selection of schools that participated in the study

Of the 50 educational centres with children with CP, 24 took part in the study. Seventeen were infant/primary schools (children aged 3–12) and seven secondary schools (ages 13–18) (Fig. 1). The sample comprised 139 children and all of them received physiotherapy in their schools.

### Ethical approval and consent

Ethics approval from Ethics Committee for Clinical Research of the University of Murcia, written informed consent of parents and the school management were obtained in all cases.

### Exclusion criteria

Individuals were excluded if they had a neuromotor disorder different from CP, if they were scheduled for upcoming surgery that would affect sitting ability, or if they were planning to move out of the area.

## Evaluation

The children were evaluated individually in the same conditions in all the centres, and the following data were recorded: 1) classification of the CP; 2) GMFCS and 3) LSS levels (Table 1). For the classification of CP, we followed the guidelines of the Surveillance of Cerebral Palsy in Europe (SCPE) [12], which classifies CP as Spastic Unilateral, Spastic Bilateral, Ataxic and Dyskinetic. The reference manual of SCPE offers a hierarchal diagnostic tree for CP and its subtypes with relatively good reliability [13, 14]. The GMFCS level was determined on usual performance in home, school, and community settings, rather than what they are known to be able to do at their best [8]. The trunk control, sitting position, postural changes and mobility of the children were evaluated in the centres by an experienced physiotherapist blinded to the study objectives.

**Table 1:** Summary of the criteria GMFCS and LSS

GMFCS	LSS
Level I	Level I
Walks without restrictions, limitations in more advanced gross motor skills	Unplaceable
Level II	Level II
Walks without restrictions, limitations walking outdoors and in the community	Supported from head downward
Level III	Level III
Walks with assistive mobility devices, limitations walking outdoors and in community	Supported from shoulders or trunk downward
Level IV	Level IV
Self mobility with limitations, children are transported or use power mobility outdoors and in the community	Supported at pelvis
Level V	Level V
Self mobility is severely limited, even with use of assistive technology	Maintains position, does not move
	Level VI
	Shifts trunk forward, re-erects
	Level VII
	Shifts trunk laterally, re-erects
	Level VIII
	Shifts trunk backward, re-erects

The LSS evaluation was made with the children sitting on a therapeutic bench with the thighs supported to the back of the knees and feet unsupported. The sitting position is assessed with the hips and knees flexed sufficiently so that the trunk is inclined at least 60°.

The surface of therapeutic bench was not too soft to affect the results.

The head may be in neutral position with respect to the trunk. The position should be maintained for at least 30 s, with due regard for the comfort and safety of the child.

As a first step in the evaluation of the sitting ability on the LSS scale, children were asked (or helped if necessary) to maintain the sitting position. If the children concerned were able to maintain their posture for 30 s, they were requested to incline their trunk and recover the original position (re-erecting the trunk), or, in some cases, they needed stimulation with a toy in addition to verbal orders to move the trunk. The children could not use their hands to keep the sitting position. The highest value achieved on the scale was recorded.

The assessments of GMFCS and LSS were carried out by an experienced physiotherapist not informed of the study objectives with almost 10 years of experience in accordance with the manuals available for both instruments [8, 16].

## Statistical analysis

A descriptive analysis of age, SCPE, GMFM and LSS were made. To assess the relation between GMFCS and LSS, Spearman’s correlation coefficient for non-parametric tests was calculated. As suggested by Cohen [17], a coefficient of <0.30 is considered a low degree of association, a value between 0.30 and 0.49 is considered moderate and >0.50 high. In order to assess the relation between each scale and CP type, a Pearson chi-square test was used. When frequency table had less than five cases, a likelihood ratio test was calculated and analysed. The level of significance was set at 0.05. All the statistical analyses were carried out using the program SPSS 18.0. (IBM Corporation, Somers, New York).

## Results

Of the 139 children, 69 (49.6 %) had spastic bilateral CP, 44 (31.7 %) spastic unilateral CP and 26 (18.7 %) were classified as dyskinetic CP. Of the cases classified as dyskinetic PC, 38.5 % were classified as choreo-athetotic CP and 61.5 % as dystonic CP. The mean age was 8.9 (SD 3.8). The minimum age was 3 years and the maximum 18.

A significant inverse relationship was found between GMFCS and the LSS levels (r_s_ = −0.86, *p* = 0.00).

The distribution of GMFCS and LSS levels is shown in Table 2. 45.3 % of the children capable of inclining in any direction and return to the neutral position (LSS VIII) could walk without limitation (GMFCS I). 8.6 % capable of inclining laterally and re-erect the trunk(LSS VII) walked with some type of limitation (GMFCS II), while 100 % of the children that could not be placed and needing support of the head, trunk and pelvis to maintain the sitting position (LSS I + II) needed a wheelchair (GMFCS V).

**Table 2:** Distribution of GMFCS and LSS levels in 139 children of the study sample

		GMFCS					TOTAL
		Level I	Level II	Level III	Level IV	Level V	
LSS	Level I	0 (0 %)	0 (0 %)	0 (0 %)	0 (0 %)	5 (3.6 %)	5 (3.6 %)
	Level II	0 (0 %)	0 (0 %)	0 (0 %)	0 (0 %)	11 (7.9 %)	11 (7.9 %)
	Level III	0 (0 %)	0 (0 %)	0 (0 %)	3 (2.2 %)	6 (4.3 %)	9 (6.5 %)
	Level IV	0 (0 %)	0 (0 %)	0 (0 %)	1 (0.7 %)	1 (0.7 %)	2 (1.4 %)
	Level V	0 (0 %)	1 (0.7 %)	3 (2.2 %)	1 (0.7 %)	2 (1.4 %)	7 (5.0 %)
	Level VI	1 (0.7 %)	3 (2.2 %)	2 (1.4 %)	2 (1.4 %)	2 (1.4 %)	10 (7.2 %)
	Level VII	0 (0 %)	12 (8.6 %)	1 (0.7 %)	4 (2.9 %)	0 (0.0 %)	17 (12.2 %)
	Level VIII	63 (45.3 %)	8 (5.8 %)	5 (3.6 %)	1 (0.7 %)	1 (0.7 %)	78 (56.1 %)
	Total	64 (46.0 %)	24 (17.3 %)	11 (7.9 %)	12 (8.6 %)	28 (20.1 %)	139 (100 %)

In relation to GMFCS, 46 % of the sample walked without limitations (Level I) and 28 % of the sample needed a wheelchair (Level V).

In relation to LSS, 56.1 % of the sample was able to incline at least 20° posterior to the vertical plane and return to the neutral position (Level VIII).

Chi-square tests revealed differences in the distribution of GMFCS levels (*χ*^2^ = 50.78) and LSS (*χ*^2^ = 37.15) and CP according to the classification of spasticity (*p* <0.01).

The distribution of GMFCS and LSS levels with the spastic unilateral CP, spastic bilateral CP, choreo-athetotic CP and dystonic CP is shown in Table 3, 4, 5 and 6.

**Table 3:** Distribution of the levels of GMFCS with the classification of SCPE (spastic CP)

GMFCS	Spastic unilateral CP	Spastic bilateral CP
Level I	81.8 %	20.3 %
Level II	15.9 %	21.7 %
Level III	2.3 %	14.5 %
Level IV	0 %	11.6 %
Level V	0 %	31.9 %

**Table 4:** Distribution of the levels of GMFCS with the classification of SCPE (dyskinetic CP)

GMFCS	Choreo-Athetotic CP	Dystonic CP
Level I	80 %	37.5 %
Level II	10 %	6.3 %
Level III	0 %	25 %
Level IV	0 %	0 %
Level V	10 %	31.2 %

**Table 5:** Distribution of the levels of LSS with the classification of SCPE (spastic CP)

LSS	Spastic unilateral CP	Spastic bilateral CP
Level I	0 %	7.2 %
Level II	0 %	11.6 %
Level III	0 %	8.7 %
Level IV	0 %	2.9 %
Level V	2.3 %	8.7 %
Level VI	2.3 %	10.1 %
Level VII	9 %	14.5 %
Level VIII	86.4 %	36.3 %

**Table 6:** Distribution of the levels of LSS with the classification of SCPE (dyskinetic CP)

LSS	Choreo-Athetotic CP	Dystonic CP
Level I	0 %	0 %
Level II	0 %	18.8 %
Level III	0 %	18.8 %
Level IV	0 %	0 %
Level V	0 %	0 %
Level VI	10 %	6.2 %
Level VII	0 %	18.8 %
Level VIII	90 %	37.4 %

**Fig 2.**
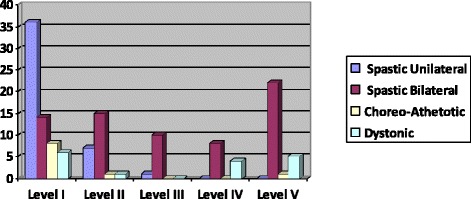
Distribution according to the SCPE with the GMFCS levels

**Fig. 3:**
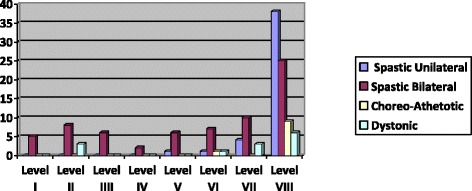
Distribution according to SCPE with the LSS levels

Figures 2 and 3 shows the distribution of the sample according to the classification of SCPE with the GMFCS and LSS levels. Of the children capable of walking with no limitation (GMFCS I), 31.9 % had spastic unilateral CP, while 19.5 % of children who needing a wheelchair (GMFCS V) had spastic bilateral CP. In the case of Diskinetic CP, 53.8 % were capable of walking with no limitation (GMFCS I) and 23.1 % needing a wheelchair (GMFCS V).

One hundred percent of the children incapable of maintaining a seated position (LSS I) and those who needed support for the head, trunk and pelvis (LSS II,III,IV) showed a bilateral spastic CP. Of the children capable of maintaining a good sitting ability (LSS VIII), 33.6 % had spastic unilateral CP. In the case of Diskinetic CP, 11.5 % of the children were incapable of maintaining a seated position (LSS I) and 57.7 % of the children were capable of maintaining a stable seated position (LSS VIII).

## Discussion

To the best of our knowledge, this is the first study to analyse the relation between the GMFCS and LSS in a sample of children with CP. For the first objective, there was a statistically significant relationship between the sitting ability and the gross motor function, finding that there was, indeed, a high degree of correlation in the 139 children of the sample. The negative value of the association reflects the fact that the scales run in opposite directions: level I of the GMFCS represents children with the greatest autonomy, while the same level on the LSS represents children with the greatest degree of dependence in sitting.

According to the level, most children able to maintain a good sitting ability (LSS VIII) and move their trunk were also capable of walking independently with or without limitation (GMFCS I,II). In contrast, children capable to maintain a seated position but not moving and those needing some sort of pelvic support (LSS IV + V) could walk but with a technical device and with limitations (GMFCS III, IV). Lastly, the children who needed support from the head (LSS II) were transported in a manual wheelchair or an electric wheelchair (GMFCS V).

Table 2 shows that the children classified in GMFCS levels I and II were capable of maintaining a seated position without support and some were capable of making some trunk movement (LSS VI-VIII). The children classified GMFCS levels IV and V showed a great variety in the ability to maintain a seated position with and without support. In our study, only 28.7 % of the total sample was classified as the last two levels of the GMFCS, which suggests that the LSS provides accurate information on the sitting abilities in children with CP.

Only in two cases children were classified in GMFCS levels IV and V and LSS level VIII. These children had spastic bilateral CP, which means that in these cases the limbs were more affected than the trunk and they were limited to the use of an electric wheelchair.

When the GMFCS was related to the classification of CP according to SCPE, there was a relation between GMFCS level and the type of CP according to the distribution of spasticity. Children with spastic bilateral CP were mainly represented as level V, a similar result to that described by Pfeifer et al. [18] and Gorter et al. [19]. In contrast, the children with spastic unilateral CP were represented as level I, which also agrees with other studies [9, 14, 20]. As far as our study is concerned, we suggest that those with bilateral CP have a greater degree of disability, generally in all four extremities, while children with unilateral CP can walk with varying degrees of limitation, possibly with technical devices. Moreover, the term bilateral includes children with spastic diplegia or tetraplegia and therefore, with different sitting abilities. It is in this point where LSS may provide more useful clinical information in terms of defining functional ability. In our study, most children with spastic unilateral CP were able to sit with a degree of stability (LSS V-VIII). In spastic bilateral CP, a greater number of subjects were distributed in different levels. The higher proportion of cases with different LSS scores in spastic bilateral CP suggest differences between the tetraplegias (low score on the scale) and diplegias (high score on the scale). Bousquet el al. [14] found that children with spastic unilateral CP predicted better sitting ability than children with other subtypes according to the classification of SCPE. However, the subtype spastic bilateral CP did not provide sufficient information as regards sitting ability in these children. In our study, the LSS enabled us to identify the sitting abilities according to the classification of the SCPE, which agrees with the results of Field et al. [6].

Whatever the case, these findings are similar to those of Carnahan et al. [21] who observed a greater limitation in gross motor skills in children with diplegia than children with hemiplegia. Gunel et al. [22] obtained results similar in children with tetraplegia. The high correlation between diplegia, tetraplegia and the GMFCS levels observed in our study supports the sensitivity of this classification to differentiate between the subtypes of spastic CP.

However, we agree with Beckung et al. [9] and Himmelmann et al. [15] in that the Swedish classification of CP types [23] is not sufficient to assess motor development, especially in diplegias, which are distributed through most of the GMFCS levels. Moreover, in clinical practice, it is difficult to differentiate severe spastic diplegia from a tetraplegia. For this reason we have used the classification proposed by the SCPE, which simplifies the terminology and diagnosis referring to children with spastic CP. Moreover, Gorter et al. [19] concluded that another subclassification according to the distribution did not increase the diagnostic value of the GMFCS. Other authors have studied the relation between subtypes of CP and the GMFCS with the presence of comorbidities, providing additional information to our knowledge of the neurodevelopment of children with CP [24].

Although Gorter et al. [20] found a low statistically significant relationship between GMFCS levels and CP according to motor impairment, we found no significant relationship between GMFCS and LSS levesl and the ataxic and dyskinetic classification according to SCPE. It seems to be uncommon to find an ataxia without dyskinetic signs and viceversa. In the case of dyskinetic CP, a greater percentage was found in the GMFCS I. Children with choreo-athetotic CP were mainly classified in GMFCS II, and children with dystonic CP were distributed across all five levels.

In the case of LSS, most of the children with dyskinetic CP (dystonic and choreo-athetotic) showed a good sitting ability with the possibility of making some kind of movement of trunk (LSS VI-VIII).

Our study was limited regarding the number of schools that participated in the study. 52 % of schools with CP children declined to participate.

## Conclusions

Our study found a negative correlation between both scales, a relation between sitting ability and the capacity to walk with or without technical devices, and the association between both scales with the distribution of spasticity according to SCPE. GMFCS and the LSS are useful tools for describing the functional abilities and limitations of children with CP based on gross motor function, specially sitting and mobility. Classification based on the distribution of spasticity and the gross motor function provides clinical information on the prognosis and development of children with CP.

## Abbreviations

CP: Cerebral palsy
GMFCS: Gross Motor Function Classification System
LSS: Level of Sitting Scale
SCPE: Surveillance of Cerebral Palsy of Cerebral Palsy in Europe
